# Biological Effects and Safety in Magnetic Resonance Imaging: A Review

**DOI:** 10.3390/ijerph6061778

**Published:** 2009-06-10

**Authors:** Valentina Hartwig, Giulio Giovannetti, Nicola Vanello, Massimo Lombardi, Luigi Landini, Silvana Simi

**Affiliations:** 1 ITENI Laboratory, CNR Institute of Clinical Physiology, Pisa, Italy; E-Mails: valeh@ifc.cnr.it (V.H.); giovannetti@ifc.cnr.it (G.G.); 2 Department of Information Engineering, University of Pisa, Italy; E-Mail: nicvanel@ifc.cnr.it; 3 MRI Laboratory, G. Monasterio Foundation and CNR Institute of Clinical Physiology, Pisa, Italy; E-Mail: lomass@ifc.cnr.it; 4 Cell Biology and Cytogenetics Unit, CNR Institute of Clinical Physiology, Pisa, Italy

**Keywords:** electromagnetic fields, Magnetic Resonance Imaging, MRI safety, genotoxic effects

## Abstract

Since the introduction of Magnetic Resonance Imaging (MRI) as a diagnostic technique, the number of people exposed to electromagnetic fields (EMF) has increased dramatically. In this review, based on the results of a pioneer study showing *in vitro* and *in vivo* genotoxic effects of MRI scans, we report an updated survey about the effects of non-ionizing EMF employed in MRI, relevant for patients’ and workers’ safety. While the whole data does not confirm a risk hypothesis, it suggests a need for further studies and prudent use in order to avoid unnecessary examinations, according to the precautionary principle.

## 1. Introduction

The human population is chronically exposed to natural and man-made sources of ionizing and non ionizing radiations, the latter being, for instance, electric and magnetic fields (EMF). Important sources of man-made radiation/electromagnetic pollution are represented by diagnostic tests. It has been reported that the medical sources of radiation amounted to about one fifth of the natural one in 1987, while only ten years later it was close to 100% [1]. Furthermore, since the introduction of Magnetic Resonance Imaging (MRI) in diagnostic examinations, the number of people exposed to EMF has increased dramatically.

While it is well established that ionizing radiations impose risks to human health and environment, not much is known about possible effects of EMF relevant for patient safety, although MRI is a diagnostic technique widely used in medicine and showing a growing impact in cardiology. Today, the great number of available MR scanners and routine clinical applications does not even allow a calculation of how many exams are performed in the world.

Quite recently, our group published a study [2] where, for the first time, we were able to show that EMF generated during MRI diagnostic scan have genotoxic effects, in terms of micronuclei (MN) induction. Although preliminary evidence suggests that an increased MN frequency is associated with early events in carcinogenesis [3], our data cannot fully confirm the presence of health hazard from MRI, as the genetic damage also seems reversible: in fact, after 48 hrs, the MN number returned to the control values, suggesting that two cell divisions are enough to eliminate lymphocyte MN.

Here, we present an updated survey of the literature on the biological/genetic effects and health implications of the electromagnetic fields present during MR scans. The three different fields (static MF, gradient MF and radiofrequency (RF) in MF are described separately: while there is a huge literature on the effects of each single type of field, only very few studies are available on their combination to generate MRI. Furthermore, we try to integrate the current findings to provide indications, mostly about occupational risk and patient safety.

## 2. Electromagnetic Spectrum

**Figure 1.**
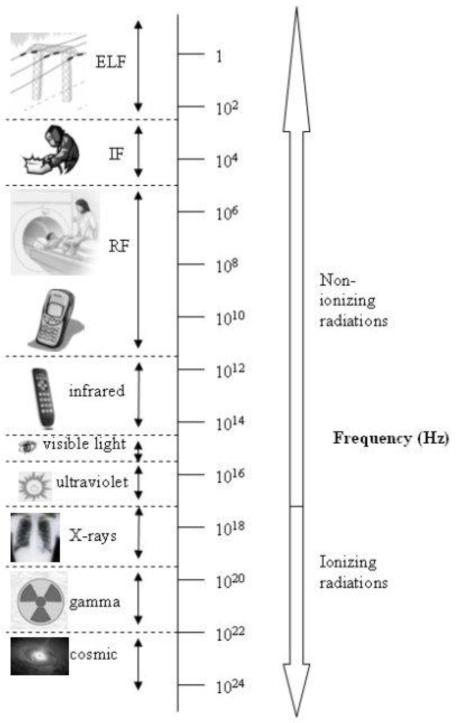
Electromagnetic spectrum and some sources of radiation.

Electromagnetic fields are classified into ionizing and non-ionizing, according to their frequency (measured in Hertz, Hz), since the ability of an electromagnetic wave to ionize an atom or molecule depends on its frequency. Figure 1 shows the electromagnetic spectrum, which extends from static field to cosmic rays, and some examples of sources. Non ionizing electromagnetic fields are classified according to their frequency in static, extremely low frequency (ELF), intermediate frequency (IF) and radiofrequency (RF) fields.

### 2.1. Static Fields

Static fields do not vary with time and are located at 0 Hz in the frequency spectrum. Typical sources of static MF are found in certain occupational settings, e.g. metal industries, welding processes and certain underground and train systems. However, the major application of high static MF is represented by Magnetic Resonance (MR), where a main magnet is used to generate a primary static field. Clinical imaging systems typically have field strengths up to 3T (1T = 10,000 Gauss, for reference the Earth’s magnetic field ≈ 0.5 Gauss) while spectroscopic systems, currently only used for research applications, are available with field strengths as high as 17.5T [4].

### 2.2. Extremely Low Frequency (ELF)

The electromagnetic fields in this frequency range (from 0 to 300 Hz), are due to residential exposure, nearby power and high voltages transmission lines and domestic installations (operating at 50 and 60 Hz) while occupational exposure sources are caused by electric power industry installations and welding devices (operating at 50 and 60 Hz). Medical applications of ELF fields include bioimpedance measurement, pain treatment and bone growth stimulation [5].

### 2.3. Intermediate Frequency (IF)

IF electromagnetic fields extend from 300 Hz to 100 kHz. Sources operating in this frequency range are anti-theft devices, typically employed for preventing theft of goods, with an exposition level which is usually below the exposure limits. Other applications are induction hobs and hotplates, electric engines and badge readers. Visual display units and some industrial applications, like induction heating and welding, also cause emissions in the IF range [5]. Typical medical applications of IF comprise gradient fields which are superimposed upon the main static field in MR applications [4].

### 2.4. Radio Frequency (RF)

RF electromagnetic fields extend from 100 kHz to 300 GHz. RF sources of this type are essentially involved in mobile communication. Mobile phones, used by more than 2 billion people all over the world, are tested by the measure of the Specific Absorption Rate (SAR), typically indicated in units of watts per kilogram (W/kg), whose maximum value should be less than 2 W/Kg for the human head, according to the guidelines of the International Commission on Non-Ionizing Radiation Protection (ICNIRP) [6]. Other wireless applications, like cordless phones or WLAN systems, operate with lower output power than mobile phones levels. The link between the mobile phone and the network is performed by base stations, which are RF transmitters, at different frequencies. Other RF sources are broadcasting (AM and FM), new digital TV technology and civil and military radar systems [5].

RF electromagnetic fields for medical application have been introduced for therapeutic and diagnostic purposes. The first group comprises soft tissue healing appliances, cancer treatment with hyperthermia, and tissue heating [5] while the second are mainly RF fields associated to MR, necessary to generate a detectable MR signal [4].

## 3. Magnetic Resonance Imaging (MRI)

During an MRI examination, three types of MF are employed to produce three dimensional images [4]: I) a high static MF, which generates a net magnetization vector in the human body, that is a measure of the proton density; II) a gradient MF (100 to 1,000 Hz), used to localize aligned protons inside the body, thus allowing spatial reconstruction of tissue sections into images; III) a RF electromagnetic wave (10 to 400 MHz), which energizes the magnetization vector allowing its detection by the MRI scanner, converting tissue properties into MR images. Different levels of contrast are based on the different magnetic properties and physical structure of the biological tissues (i.e. density of hydrogen atoms) [4].

The major recognized mechanical risk associated with MR scanner is the presence of ferromagnetic devices and equipments, including biomedical implants. These equipments will be subject to the attractive (projectile effect) and rotational forces, caused by the static field, whose magnitude depends on their mass and distance from the bore entrance [4].

The projectile effect caused the most serious accident reported to date: a 6-year-old boy died after an MRI exam, when the machine’s powerful MF jerked a metal oxygen tank across the room, crushing the child’s head [7]. Other accidents have been related to thermal injuries that usually occur where the skin is in contact with a monitoring sensor or cable [8,9].

Cardiovascular MRI is an increasingly adopted modality for the evaluation of patients with cardiovascular diseases. Potential hazards are associated with the presence of cardiac devices and implants, such as heart valve protheses, coronary artery stents, aortic stent grafts, pacemakers and implantable cardioverter-defribrillators, due to possible movement, dislodgment, dysfunction or damaging of the cardiac device caused by the interactions with the MF [10].

### 3.1. MRI Biological Effects

Magnetic Resonance Imaging is considered a safe technology since it just has the ability to change the position of atoms, but not to alter their structure, composition, and properties, as the ionizing radiations attempt to do. However, as in any sanitary interventions, there are intrinsic hazards that must be understood, acknowledged and taken into consideration. These hazards are relative to all three types of fields which can affect patients, staff and other persons within the MR environment [11].

To assess the potential dangerous biological effects associated with MRI environment and procedures, several studies have been conducted over the past thirty years, often producing controversial results.

Most of these studies are relative to the biological effects of a particular electromagnetic source utilized in MRI, while there is a lack of knowledge about the combination of three MF components. Thus, there is a need to integrate the current findings to better understand the interactions between EMF related to MRI and biological systems.

This review is divided in three sections, according to the three sources of EMF utilized in MRI procedures. In each section, the risk assessment related to each field component is summarized. We focus only on mammalian/human biological systems for their obvious strict correlation with human health.

#### 3.1.1. Effects of Static MF

The safety of static MFs has been discussed for more than a century: in 1921 Drinker and Thompson [12] carried out numerous experiments to investigate possible effects on workers exposed to MF in industrial applications. They concluded that the static MF had no significant hazard effects on human health.

More than 400 papers have been published on the biological effects of static MF, but the results were often contradictory and confusing [13].

With the advent of MRI at the beginning of the eighties, the interest in understanding the potential hazards associated with static MF exposure has increased. A recent review concluded that it was very difficult to prove the existence of significant biological effects of static MF [14], with the exception of force orientation effects on biological molecules with particular magnetic properties (i.e. haemoglobin, free radicals), without apparent side effects for humans [15], and some sensory effects such as nausea, vertigo and metallic taste [16].

A recent paper of ICNIRP reviewed *in vivo* and *in vitro* studies carried out to detect biological responses to static MF in the range of milli T up to several T, in order to give new guidelines on limits of occupational exposures and exposure of general public [17]. The new proposed values are 2T for the occupational exposure of head and trunk, 8T for the occupational exposure of the limbs and, finally, 400mT for the general public exposure of any part of the body. These new guidelines do not apply to patients undergoing medical diagnosis or treatment: detailed considerations on the protection of patients are in preparation.

##### - *In vitro* effects

Many studies have been carried out on the *in vitro* effects of static MF. Cell growth, cell proliferation, cell cycle distribution pattern and apoptotic cell death seem not to be affected by an exposure up to four days at field strengths up to 10T [18], while an exposure of 10-17T for 30–60 minutes can reduce number and size, cells organization and vitality as observed in cultured mammalian cells [19]. A blood oxygenation dependent increase in blood viscosity due to an exposure of 1.5T was also observed in [20].

Genotoxic or carcinogenic effects have also been studied [21] and it was suggested that static MF might affect the process of cancer induction and/or progression by altering cellular responses to some known carcinogens (chemicals, radiation). In any case, the body of results available in the literature are often not comparable and in some cases also not reproducible making a definitive conclusion premature.

##### - *In vivo* and *ex vivo* effects

###### Mammals

Various experimental studies carried out over the last 30–40 years have examined the effects of chronic or acute exposure of laboratory animals to static MFs. Four main areas of investigation have been covered: nervous system and behavioural studies, cardiovascular system responses, reproduction and development, and genotoxicity and cancer.

No effects were found on neurophysiological responses (ion channel conduction properties, nerve conduction velocity, excitation threshold) in rats, cats, monkeys and frogs after an exposure at static MFs of up to 2T [22,23].

Neurobehavioral studies have shown a lack of effects on the normal activity of animals under exposure up to 1.5T, while exposures higher than 1.5T have led to adverse responses [23].

A change in Na^+^ or K^+^ ion channel conductivity produced by an exposure at 24T [24], and a reduction of visual evoked potential in the cat brain following an exposures to 120mT for 150s [25,26] were reported. It was suggested [27] that these effects result from the slow re-orientation of aligned groups of diamagnetic phospholipid molecules within the cell membrane.

Effects on cardiovascular function, including arterial blood pressure and peripheral blood flow, are less clearly established [22,28].

Few studies have examined the effect of static MFs on reproduction and development: there are generally no effects by exposure up to 9.4T, but the studies showed several inconsistencies [29].

Also, sub-chronic exposure (10 weeks to a 9.4T static MF) seems to have no biological effects (alterations in heart rates, body weights, food and water consumption, blood biochemical and urinary parameters and major organ weights) in male and female adult rats or their progeny [29].

It is generally accepted that static fields below 1T are not genotoxic [30,31]. However, a recent study [32] reported significant, time and dose-dependent increases of the micronuclei frequency in mice exposed to static MFs of 2, 3 or 4.7T. Again, the general consensus is that there are insufficient studies to draw any conclusions relative to the genotoxicity or the carcinogenicity of static MF [33].

###### Humans

Studies on human volunteers exposed up to 8T, carried out to assess information about the relationship between exposure to high static MFs and human health, took into account as endpoints central and peripheral nervous activities, behavioural and cognitive functions, sensory perception, cardiac function, respiratory frequency, body temperature, but no conclusion could be drawn [15].

Temporary and dose-correlated vertigo and nausea in workers and patients exposed to static MFs higher than 2T have been found in several studies [34,35], while the correlation between the exposure and the metallic taste has not been confirmed [35]. No significant differences among several physiological parameters (heart rate, blood pressure, blood oxygenation, core temperature, ECG, respiratory rate) have been checked during the exposure at 8T, together with complete reversible tachycardia imputable to the stress correlated with the exam [34].

Finally, acute neurobehavioral effects, such as eye–hand coordination speed and visual and auditive working memory problems after exposure to static fields at 1.5 and 3T have been reported for health volunteers in [36].

A non statistically significant increase in the number of spontaneous abortion of MRI workers has been reported [37]. Different effects, such as fertility, length of gestation, birth weight, pregnancy outcome and offspring gender for pregnancies exposed to the MRI have also been reported [38], but these studies present methodological limitations and cannot be considered conclusive.

A recent review summarizes the epidemiological evidence of static MF exposure and long-term health effects: the few studies available have focused on cancer risks and the results from these studies are not sufficient to draw any conclusions [39].

Finally, the available data do not allow one to reach a firm conclusion about the health effects of the static MF [16].

*A document of the World Health Organization (2006, [15]), stated that there are no evidences on the short and long term adverse effects of the MRI static MF on human health. This statement has been confirmed also more recently* [16,17]. *Considering the increased use of MR scanners with higher static MF values, there is an urgent need to perform studies to provide assurance about their safety.*

#### 3.1.2. Effects of Gradient MF

During an MRI examination, the gradient MF, which serves for the spatial localization in the image reconstruction process, is often switched on and off. For this reason, they are considered time varying MFs ranging between ELF and IF. Most of the available studies deal with possible association between residential ELF and cancer [33]. ELF MF has been classified in group 2B (“possibly carcinogenic to humans”), due to the possible association between residential MFs and childhood leukaemia [40]. Furthermore, a decreased survival of children with leukaemia after exposure to ELF magnetic fields has been observed [41], while no correlations have been established between ELF field exposure and breast cancer risk [42].

The time variation induces in the patient undergoing a MR scan, an electric field which could stimulate nerves and muscles, and could generate cardiac stimulation or even ventricular fibrillation. While the latter is a primary concern, being a life-threatening condition, possible peripheral nerve stimulation may cause discomfort and could not be tolerated by the subjects, thus interfering with the examination (e.g. due to patient movements) or would result in a request to stop the examination [43].

Due to technical difficulties for obtaining a reliable measure of induced electric currents, several works now are dealing with numerical simulations in human models [44].

##### - *In vitro* effects

A significant increase of DNA strand breaks after ELF exposure was reported [45], while non-genotoxic mechanisms, such as stimulation of cell proliferation and apoptosis inhibition, can act as environmental agents for promoting cancer development [46]. An increase of micronucleus frequency in human fibroblasts exposed to a 50 Hz power line signal has been reported [47].

Mouse cell cultures exposed to gradient fields for hours did not show any effects with gradient fields of 25 mT/m in 300 ms [48]. Other studies [49,50] report no significant genotoxic effects as measured by sister chromatid exchange frequencies in human lymphocytes exposed to time varying fields of up to 220 μT, suggesting they are unlikely to act as carcinogens. Other studies report increased DNA synthesis in human fibroblast with exposure at 4 to 15 kHz [51], while fetal cell growth and cell cycle distribution of human lung fibroblasts exposed to gradient of 10mT/m are not affected [52]. These results provided no support for a teratogenic effect of this type of MF.

Detrimental effects of co-exposure to ELF and environmental carcinogens are reported, such as recombination of radical pairs alterations, indicating interactions among MF and chemical and/or physical agents [53]. This fact suggests that human population could be exposed to a variety of environmental insults which may not be genotoxic “*per se”*, but they may enhance the negative effects induced by other contaminants [2].

##### - *In vivo* and *ex vivo* effects

*In vivo* studies on gradient MFs mainly deal with the importance of determining a threshold value and aim to understand any possible carcinogenic potential.

###### Mammals

It was observed that dogs’ peripheral nerves are more sensitive to gradient MF, thus showing the lowest stimulation threshold [54]. In other studies, it was observed that the threshold for respiration was three times more than the peripheral nerve stimulation threshold, while the cardiac one was about nine times greater than the peripheral nerve one [55,56]. Studies on reproduction and development of mammals showed that these parameters are not affected by IF field exposure [57].

###### Humans

A 2000’s review [58] analyzed patient safety in time-varying gradient fields associated to a MR scan and concluded that cardiac stimulation is very unlikely in present-day systems, while at sufficient amplitudes, peripheral nerve stimulation is perceptible (tingling or tapping sensations) and can cause patient discomfort. Current safety standards have been developed by the International Electrotechnical Commission [59], establishing that the threshold for cardiac stimulation is largely above the value causing peripheral nerve stimulation, thus avoiding subjects’ ventricular fibrillation [60].

The use of MRI gradient MF represents a potential health risk beside peripheral nerve and cardiac stimulation to the patient. With the advent of the new generation of MR systems characterized by higher static MF and faster gradient fields, their effects on human health should be the object of further and properly designed studies.

#### 3.1.3. Effects of RF Fields

During an MRI scan, the patient is exposed to a time varying electromagnetic field in the RF range. It has been suggested that RF can induce effects via multiphoton absorption, i.e. through direct heating [61].

Thus, biological effects caused by RF field can be classified into two categories [62]:

- non thermal effects: due to direct interactions between MFs and tissues
- thermal effects: due to tissue heating caused by the induced electric currents

The non thermal effects have been less studied, however adverse effects mainly arise from a direct energy transfer from the field to the living system, which might be strongly non linear, and are dependent on the field frequency [63].

The temperature increase of the tissues due to the RF energy absorption, depends on parameters such as the electrical and geometrical tissue properties, the type of RF pulse used, its repetition time and the frequency of the radiation. The frequencies generally used in a MRI scanner are in the range at which high absorption occurs in the whole body [6]. Certain organs, such as the eyes and testes are, particularly sensitive to heating due to lack of perfusion, so the presence of “hot spots” at those sites can be very dangerous for the patient safety [64,65].

Moreover, tattoos and permanent cosmetics realized with iron oxide or other metal-based pigments, can cause reactions or adverse events (including first and second-degree burns) [66–68].

The dosimetric parameter, normally used in safety standard and guidelines to quantify the energy absorption caused by RF, is the SAR [69]. During a MR scan the patient’s temperature is not easy to measure so SAR represents a convenient parameter to control any possible temperature increases. Generally, the MRI scanner software allows monitoring of the SAR for the whole body: these values have to be always below the limits values set by IEC standard [59] and must be recognizable by the software, so that if the SAR value exceeds the standard limits, the software stops the scanning process. The admitted SAR is usually 4 W/kg for a whole body scanner, calculated for a body temperature increase up to a 0.6 °C and a scanning period of 20–30 min [70,71].

It has been reported that, while average whole body SAR remains below the safety limits [72,73], hot spots could occur all the same making the automatic control system of the scanner not totally sufficient to assure patient safety.

##### - *In vitro* effects

There is a very wide body of literature regarding the possible induction of toxicity, genotoxicity, and transformation on mammalian cells *in vitro* due to high RF fields employed in cellular telephones (900-1,100 MHz) [74]. Although it is well known that the radiation energy from mobile phones is much lower than the energy necessary to break chemical bonds, several authors have reported DNA strand breaks, micronuclei induction and chromosomal aberrations [75] in human fibroblasts. Transient increase of DNA strand breaks in embryonic stem cell have also been reported [76].

An attempt to independently replicate those results with the same biological system, under the same RF exposure, failed and negative results were obtained [77]. Cell cycle kinetics [78] and apoptosis induction [79] has been reported to be unaffected.

It has been also investigated whether 24 h exposure to RFs, similar to those emitted by mobile phones, could affect micronuclei frequency and cell proliferation in cultured human peripheral blood lymphocytes: no evidence of genotoxicity or cytotoxicity was found [80].

Furthermore, possible effects related to the third generation wireless technology (1,950 MHz Universal Mobile Telecommunication System, UMTS) were investigated in human. The results indicated that both long and short duration intermittent exposures induce neither an increase in micronucleated cells, nor changes in cell cycle kinetics [81].

Studies on effects due to RF at frequencies related to MR procedures are far less available. The whole data on mammalian cell cultures [82] suggest that RF exposure does not cause an increase in gene mutation, in chromosome aberration frequency or in sister chromatid exchange frequencies, suggesting that RF exposure during a MR procedure is unlikely to be genotoxic.

Similar results, using the same biological endpoints, are obtained treating human lymphocytes [83].

To exclude thermal effects, in our study [2] the temperature of the liquid in the flasks was continuously monitored using a fiber optic temperature sensor: the observed thermal increase observed was always below 1 °C. This increase is known to be under the risk level, provided the thermoregulatory function of the patient undergoing MRI scan is not compromised [28].

##### - *In vivo* and *ex vivo* effects

###### Mammals

Several studies have been carried out on animals to determine thermoregulatory reactions to tissue heating due to RF radiation at typical MR frequencies. These experiments demonstrated that RF exposure can cause a body temperature increase [63]. However, the results from animals cannot be extrapolated directly to humans since the pattern of the RF absorption strongly depends on the body size, the anatomical features and the sensitivity of the tissues [11].

###### Humans

As for *in vitro* experiments, most of the *in vivo*/*ex vivo* data on RF effects are related to mobile phone frequencies. The possible association between RF exposure due to mobile phone use and cancer has been largely subjected to epidemiological studies. Most of these studies found no association [84,85], while only a few suggested possible links [86]. Data on cancer induction, mainly intracranial tumours, are contradictory [87,88].

The cancer risk related to RF fields generated by television and radio transmitters was also analysed [89]: no study has confidently suggested any clear links to health effects [90].

Due to the increasing use of mobile phone by even young children, one important issue is related to and the question is about the possible differences in RF absorption between children and adults during the use of mobile phone [91].

Few studies have addressed the correlation between RF field exposure and the so-called “electromagnetic hypersensitivity”, which includes non-specific self-reported symptoms (headaches, fatigue, concentration difficulties). The data suggest that these symptoms cannot be correlated to RF exposure [92].

The first experiment on human thermal response to RF during a MR procedure was performed in 1985 [93]: in subjects exposed to a SAR value equal to 4 W/kg, the temperature changes and other physiological parameters, such as heart rate, were monitored. No abnormal temperature increase or changes in physiological parameters were observed. Other studies on volunteers have always reported changes in body temperature of less than 0.6 °C, without alterations in parameters like heart rate, blood pressure and blood flow [94,95].

Another study on volunteers [96] exposed to MR procedures with a high whole body SAR value (6 W/kg) monitored tympanic and skin temperature, heart rate, blood pressure, oxygen saturation and skin blood flow: statistically significant changes where found in some parameters such as skin blood flow, systolic blood pressure and heart rate, but all these changes were within acceptable safety levels.

A 2000’s review summarized physiological alterations in visual, auditory, endocrine, neural, cardiovascular, immune, reproductive, and developmental functions, under RF exposure: high levels of exposure were found to be related to an alteration of these functions [63].

Special attention was paid to over-heating of gonads [64] and eyes [65,97] for their reduced capabilities of heat dissipation thus becoming possible hot spots. In these experiments the observed temperature, however, was always below the recognized safety thresholds.

To date there have been no epidemiological studies regarding RF fields associated with MR procedures. The ICNIRP therefore recommends epidemiological studies to be done on subjects with high levels of cumulative exposure or with particular conditions, like pregnant occupational workers. Because of the advent of new generation MRI scanners with higher MFs, there is an urgent need for monitoring workers [98].

*Interactions between RF and biological tissues during MR procedures could be unsafe for patients [11]. Most of the reported accidents are burns due to hot spots in presence of conducting materials close to the patient such as the leads of physiological parameters (heart rate, blood pressure, oxygen saturation and temperature) monitoring equipment. This kind of risk can be more serious in case of internal biomedical implants (aneurism clips, stent, etc) especially for implants that have elongated configurations and/or are electronically activated (neurostimulation systems, cardiac pacemakers) [10,99,100]*.

*MRI generated RF are unlikely to be genotoxic, but unfortunately, to date no epidemiological studies are available to assess possible long term health effects due to these radiations*.

#### 3.1.4. Effects of combination of Static, Gradient and RF fields during MRI scan

During a MRI scan, patients are exposed to combinations of static, gradient and RF fields. Besides minor adverse events, such as nausea and rare allergic reactions or tissue necrosis, associated with containing-gadolinium-contrast agents used routinely for MR examinations [101], more relevant for human health are the effects on biological parameters. Unluckily, very few works deal with the biological effects due to the simultaneous exposure to the three types of MF.

The cell cycle progression was studied in human cell lines under conditions similar to MRI clinical routine exams and no alterations were observed [102]. The effects of long duration high field MRI on fetal growth and postnatal development of mice were also studied in [103], without any statistically significant changes being observed.

Recently, some biophysical properties of erythrocytes were analyzed in 25 patients during a MRI scan [104]. The results showed a significant decrease in red blood cells membrane permeability, membrane elasticity and erythrocytes sedimentation rate during MRI, but the removal of the MF resulted in a rapid return to the normal conditions.

In our work, the possibility that MRI tests could be associated to DNA damage was investigated by *in vitro* as well as *in vivo* experiments [2]. Experiments were carried out both *in vitro*, by exposing lymphocyte cultures from healthy subjects to MRI for different periods and different variable magnetic fields (MFs) obtaining dose-effect curves, and *in vivo*, analyzing lymphocyte cultures set up from individuals before and after cardiac MRI scan. Statistically significant induction of MN was found consistently both *in vitro* and *in vivo* experiments. A certain degree of repair of the genetic damage across time was also observed. This former result is quite relevant for patient’s safety: after 48 hrs, the MN numbers returned into control values, suggesting that two cell divisions are enough to eliminate all MN from the lymphocytes population. This short recovery time may be due to death of micronucleated cells or to their dilution in the pool of unaffected dividing cells. In the *in vivo* experiments we used a clinical protocol for cardiac examinations but we consider our results to have a general impact for all MR procedures.

The observed increase of the MN frequency, followed by a rapid return to normal values, although not confirmative of a hypothesis of risk for people undergoing MRI examinations, strongly suggests the need for further studies.

### 3.2. Occupational Risk

The staff operating in the environment of MRI scanners is exposed daily for hours to essentially static MFs, as the other two kind of radiation, gradients and RF, are present only inside the scanner. However, when the clinical needs force them to move close to the scanner during the examination, they could also be exposed to the other two types of radiation. These concerns have also been raised recently [16,105] and to protect occupational workers, European Union has required to incorporate the physical agents directive (PAD) 2004/40/EC [106] into its legislation, which, in some cases, could restrict the use of MRIs.

A 2008’s review summarized studies on health effects of occupational exposure to static MFs [16]: with the available data no firm conclusions can be drawn about these effects. According to the Directive 2004/40/EC [106], the workers exposed to MF should receive all necessary information about the potential risks; due to the uncertainties resulting from the available evidence, it is needed ability to find a balance between few certainties and several doubts.

Our work [2] also pinpointed the relevance of (sub)chronical exposure: during the *in vitro* experiments we used control flasks located in the console room, and other flasks, named as “room controls”, located in the scanner room, around three meters far from the scanner bore. These flasks were exposed to 1 Gauss static MF and to negligible RF and gradients fields. Room control data showed no statistically significant differences, even though a weak increase was always observed (data not shown). This observation suggests a need of awareness on occupational risk assessment for MRI operators, but also for the general population that could be exposed to different environmental insults, not genotoxic “*per se”*, which may enhance the negative effects induced by other biological, chemical and/or physical agents.

## 4. Concluding Remarks

While it is well established that ionizing radiations impose risks to human health and the environment, the available data on possible effects of MRI procedures relevant for patient and worker safety are not sufficient to draw any conclusions. For this reason, in 2003, the FDA declared “nonsignificant risk status” for MRI clinical systems generating static fields up to 8T [107].

To our knowledge, our work [2] has been the first, and up to now the only one, demonstrating any genotoxic effects induced by MRI scans. We concluded that better auditing rules and a more informed consent will reduce the number of inappropriate examinations, thus avoiding detrimental effects both for public health and environment, it being understood that MRI procedures are relative more safe than any other clinical test using ionizing radiations. Anyway, until a wider knowledge of the potential risk related to diagnostic MRI is available, a prudent attention should be adopted in order to avoid unnecessary examinations, according to the precautionary principle.

In recent time, the importance of citizen/patient involvement at all levels of the health services is increasingly recognised as a useful and positive value. Involving patients in health care decisions promotes greater patient responsibility which ultimately leads to improved health outcomes [108]. Professional/patient shared decisions could also help to avoid over-prescriptions: this will also help to cut inappropriate and unnecessary costs in a world where resources tend dramatically to finish and healthcare systems are struggling to provide necessary basic care to their populations.

Recently, the New York Times [109] stated that what makes Americans sick is an “epidemic of diagnoses”, that leads to an epidemic of treatments which turns ordinary people into patients. Education of the public and the provision of good quality information is becoming vital: the new challenge for the NHS is to give the way for active public participation and empowerment. This is the road map for an accountable growth of the health system.

## Figures and Tables

**Figure 2.**
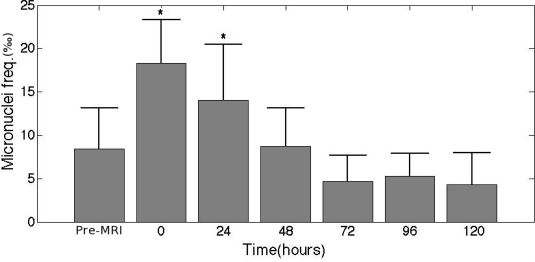
MN induction at different times after cardiac MRI scans. * Statistically different from control (p < 0.001)
